# Crack-Length Estimation for Structural Health Monitoring Using the High-Frequency Resonances Excited by the Energy Release during Fatigue-Crack Growth

**DOI:** 10.3390/s21124221

**Published:** 2021-06-20

**Authors:** Roshan Joseph, Hanfei Mei, Asaad Migot, Victor Giurgiutiu

**Affiliations:** 1 Department of Mechanical Engineering, The University of Texas at San Antonio, San Antonio, TX 78249, USA; roshan.joseph@utsa.edu; 2 Department of Mechanical Engineering, University of South Carolina, Columbia, SC 29208, USA; hmei@email.sc.edu; 3 Department of Mechanical Engineering, College of Engineering, Thi-Qar University, Nasiriyah 64001, Iraq; migotasaad@gmail.com

**Keywords:** structural health monitoring, SHM, fatigue cracking, acoustic waves, crack length detectability, crack resonances, piezoelectric wafer active sensors, PWAS, FEM analysis

## Abstract

Acoustic waves are widely used in structural health monitoring (SHM) for detecting fatigue cracking. The strain energy released when a fatigue crack advances has the effect of exciting acoustic waves, which travel through the structures and are picked up by the sensors. Piezoelectric wafer active sensors (PWAS) can effectively sense acoustic waves due to fatigue-crack growth. Conventional acoustic-wave passive SHM, which relies on counting the number of acoustic events, cannot precisely estimate the crack length. In the present research, a novel method for estimating the crack length was proposed based on the high-frequency resonances excited in the crack by the energy released when a crack advances. In this method, a PWAS sensor was used to sense the acoustic wave signal and predict the length of the crack that generated the acoustic event. First, FEM analysis was undertaken of acoustic waves generated due to a fatigue-crack growth event on an aluminum-2024 plate. The FEM analysis was used to predict the wave propagation pattern and the acoustic signal received by the PWAS mounted at a distance of 25 mm from the crack. The analysis was carried out for crack lengths of 4 and 8 mm. The presence of the crack produced scattering of the waves generated at the crack tip; this phenomenon was observable in the wave propagation pattern and in the acoustic signals recorded at the PWAS. A study of the signal frequency spectrum revealed peaks and valleys in the spectrum that changed in frequency and amplitude as the crack length was changed from 4 to 8 mm. The number of peaks and valleys was observed to increase as the crack length increased. We suggest this peak–valley pattern in the signal frequency spectrum can be used to determine the crack length from the acoustic signal alone. An experimental investigation was performed to record the acoustic signals in crack lengths of 4 and 8 mm, and the results were found to match well with the FEM predictions.

## 1. Introduction

Due to the growing number of aging engineering structures and variable working conditions, an effective technology for health monitoring purposes is required from the scientific community. The AE analysis method is a well-known structural health monitoring (SHM) and non-destructive testing (NDT) technique. The AE analysis method has been used for passive sensing of acoustic signals during damaging processes, including impact damage, fatigue-crack growth, and plastic deformation, in metallic structures. Fatigue-crack growth is a common problem in metallic structures. The severity of fatigue-crack growth increases with the age of the metallic structure. The fatigue-crack growth in metallic structures generates AE signals due to the formation of cracks. Many researchers have identified the characteristics of AE signals during fatigue-crack growth using various sensors. However, little research has been performed regarding the correlation between the crack length and the AE signal signatures. The exact quantification of the crack length is essential for scheduling the maintenance of the structure in which the crack growth is occurring. In this research, a novel method was introduced to evaluate the AE fatigue-crack length from the AE signals recorded during fatigue-crack growth using a single PWAS sensor.

The AE technique has been used for damage detection and source localization of fatigue-crack growth in metallic structures. The AE method is a passive, wave-propagation-based SHM method for in situ monitoring. The study of acoustic emissions during a fatigue-crack growth event has attracted significant research attention. Numerous researchers have studied the AE caused by fatigue-crack growth, in addition to wave scattering from fatigue cracks [1,2,3,4,5,6]. Zhang et al. [7] studied the acoustic emission signatures of fatigue damages in an idealized bevel gear spline and identified two different AE signal signatures for plastic deformation and a crack jump. Bhuiyan et al. [8,9,10] studied the AE signal signatures recorded by PWAS transducers during a fatigue-crack growth experiment in thin metallic plates. In this research, under a slow frequency of fatigue loading (<0.25 Hz), the AE signals were recorded for a short advancement of crack length, and eight signal signatures were identified related to crack growth, and crack rubbing and clapping. Hamstad and McColskey [11] studied the detectability of slow crack growth AEs in a fatigue experiment. In this research, a 0.3 Hz cyclic fatigue loading was applied to specimens of steel and aluminum with a thickness of 1 inch. In the experiment, the crack growth rate was controlled by maintaining a fixed range of the stress intensity factor (ΔK) with set minimum and maximum loads. This method achieved very slow fatigue-crack growth rates of 1 × 10^−4^ mm/cycle and 1 × 10^−3^ mm/cycle. Four resonant sensors and four wideband sensors were used to record the AE signals. Via waveform inspection, crack events were separated from grip events. It was found that the use of wideband sensors can enhance the ability to make the necessary distinction of AE signals during a fatigue experiment. Roberts and Talebzadeh [1] discussed the correlation between acoustic emission count rates and crack propagation rates. Various signal processing methodologies and clustering techniques have been used to differentiate AE signals caused by various activities [12]. Correlations between crack characteristics and AE signal features have also been analyzed using different techniques [13,14,15,16]. Recently, a novel Savitzky–Golay filter (SGF) was used for damage location and quantification in a bridge under a moving load [17]. A vibration-based structural damage identification method for structures under the influence of temperature and noise was developed by Huang et al [18]. Various techniques have been developed to detect damage in engineering structures based on the interaction of waves with damage [19,20,21,22,23]. Researchers have effectively used clay boundaries to prevent the reflection of waves from the plate boundaries during ultrasonic experiments. Clay boundaries have been used for reducing reflections from AEs during a low cycle fatigue experiment [8]. A FEM numerical study was also conducted of the effects of providing damping at the boundaries of a plate to prevent boundary reflection [24].

Numerous researchers have performed theoretical and numerical studies of AEs, thus providing an understanding of the correlation between AE and AE-source mechanisms. Ohtsu and Ono developed a generalized theory of AE and AE-source mechanisms in half-space [25] and a theoretical investigation of tensile and shear cracks in half-space [26]. Finite element modeling of an AE source using dipole and monopole source definition and simulation was also performed to study the AE signals [27,28,29,30]. In another study, the AE was coupled with direct particle observation to relate AE features to particle fractures during cyclic loading conditions [31].

Various AE sensors have been used to detect AE signals; in particular, we gave used R15a [32] and S9225 [33] from Physical Acoustics Corporation. The R15a is a resonant sensor with a resonance frequency of 150 kHz, whereas the S9225 is a wideband sensor with a comparatively small size. Various types of resonant and wideband AE sensors are currently available. Fiber optic sensors, such as fiber Bragg grating (FBG) AE sensors, have high sensitivity and small size. However, the FBG sensor performance is affected by the sensor’s directional sensitivity [34,35,36]. Piezoelectric wafer active sensors (PWAS) are commonly used to detect AE signals [37,38]. PWAS transducers have excellent characteristics, such as high sensitivity and good stability [39].

The essence of this study was to determine that the crack length can be detected from the AE signals originating at the crack tip during crack growth. In this research, we used a PWAS sensor to sense the fatigue-crack growth AE signal. The AE signals caused by crack growth interact with the crack and, following the interaction, the AE signals reach the sensor. For different crack lengths, the AEs interact differently with the crack, and the resulting AE signal sensed by the sensors is different. In the present study, numerical and experimental investigation of the fatigue-crack AE was undertaken to determine the effect of crack length on AE signal. The organization of this paper is as follows. In Section 2, a closed-form expression of AE signal sensing using the PWAS sensor is derived. Section 3 presents the FEM simulation of fatigue-crack growth AE excitation, which was performed using the concept of dipole moment excitation source. Using the FEM wavefield and theoretical equation of PWAS sensing, the prediction of the AE signal sensed by PWAS caused by the growth of fatigue cracks with lengths of 4 and 8 mm, from tip to tip, was performed. The frequency spectrum of the AE signal sensed by the PWAS sensor was studied. A proportional change in the peak–valley pattern of the AE signal’s frequency spectrum was observed due to the change in crack length. Section 5 presents the experimental measurement of the fatigue-crack growth AE signal for crack lengths of 4 and 8 mm, which was performed using a novel stress intensity factor (SIF)-controlled fatigue experiment. A good match of experiment and simulation was observed. The paper ends with a summary, conclusion, and scope for future work.

## 2. PWAS Sensor and Sensing Equation

**Figure 1:**
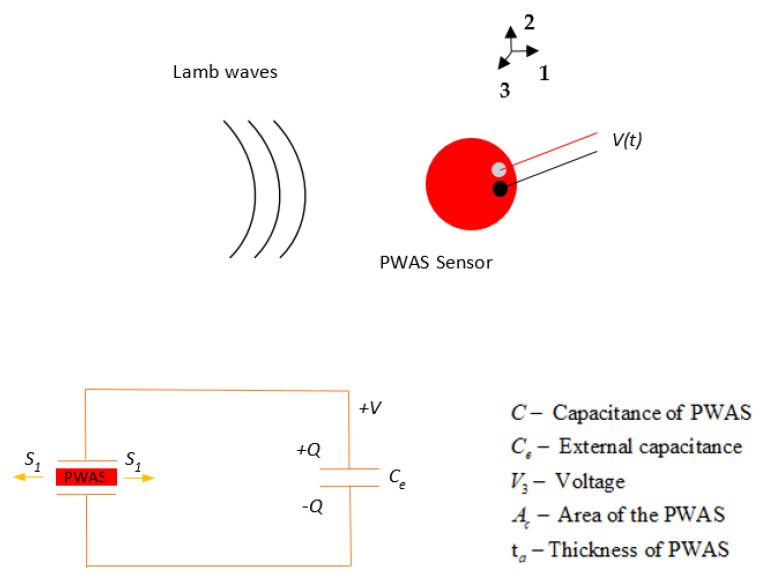
PWAS sensing mechanism schematic for Lamb wave detection.

Piezoelectric wafer active sensors (PWASs) are inexpensive, lightweight, small, and unobtrusive transducers that work on the piezoelectric principle [40]. PWASs are available in different geometries and dimensions. They can be permanently bonded to a structure to transmit and receive guided waves. The PWAS transducer couples the electrical and mechanical effects. The PWAS sensing mechanism is presented in Figure 1. The PWAS senses the in-plane and out-of-plane wave motions by sensing the in-plane strain. The method of operation of the PWAS transducer element depends on the polarization of the lead-zirconate-titanate (PZT) during the manufacturing process.

For a piezoelectric material polarized in the *x*_3_ direction, the constitutive piezoelectric equations are [41]:

(1) $$\begin{array}{l} S_{1}=s_{11}^{E}\sigma_{1}+s_{12}^{E}\sigma_{2}+d_{31}E_{3}\\ S_{2}=s_{21}^{E}\sigma_{1}+s_{22}^{E}\sigma_{2}+d_{32}E_{3}\\ D_{3}=d_{31}\sigma_{1}+d_{32}\sigma_{2}+\varepsilon_{33}^{T}E_{3} \end{array}$$

where:

$\begin{array}{l} S_{p}—\;\mathrm{Strain}\;\mathrm{on}\;\mathrm{the}\;\mathrm{PWAS} \end{array}$
$\begin{array}{l} s_{pq}^{E}—\;\mathrm{Compliance} \end{array}$
$\begin{array}{l} \sigma_{q}—\;\mathrm{Stress}\;\mathrm{on}\;\mathrm{the}\;\mathrm{PWAS}\; \end{array}$
$\begin{array}{l} d_{iq}^{},d_{kp}^{}—\;\mathrm{Electrical}-\mathrm{mechanical}\;\mathrm{coupling} \end{array}$
$\begin{array}{l} \varepsilon_{ik}^{T}—\;\mathrm{Electric}\;\mathrm{permittivity} \end{array}$
$\begin{array}{l} E_{k}—\;\mathrm{Electric}\;\mathrm{field} \end{array}$

Simplifying Equation (1) for the voltage sensed by the PWAS, we obtain:

(2) $$V=\frac{1}{C_{e}+\left(1-k_{p}^{2}\right)C}\frac{d_{31}}{s_{11}^{E}(1-\upsilon )}\int_{A_{c}}(S_{1}+S_{2})dA$$

In this research, a PWAS sensor with a diameter of 7 mm and thickness of 0.5 mm, polarized in *d*_31_ operation mode, was used for AE sensing. Thus, Equation (2) was the analytical equation used to obtain the voltage sensed by the PWAS from the in-plane strain.

## 3. FEM Analysis of Fatigue-Crack AE

**Figure 2:**
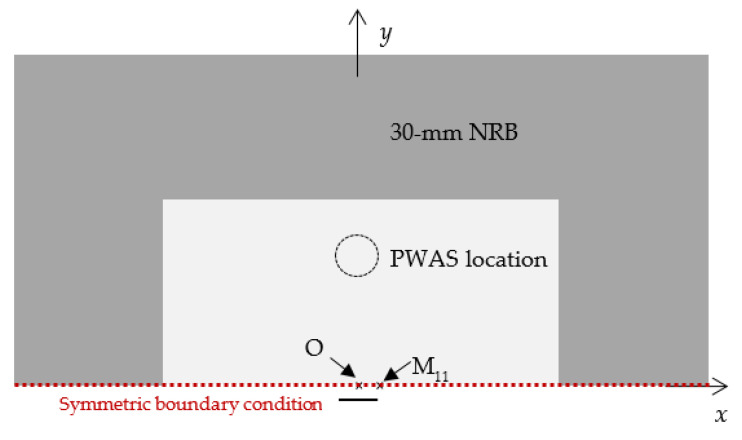
Simplified FEM model using the symmetric boundary condition for the AE simulation.

A FEM analysis was carried out to investigate the correlation between fatigue-crack growth AEs and crack length during a fatigue-crack growth event. The ANSYS software package was used to build a 3D model with length of 120 mm, width of 60 mm, and thickness of 1 mm. A half model was developed because the symmetric boundary condition was used to reduce the computational time. The material properties of aluminum 2024-T3 (73.1 GPa Young’s modulus, 0.33 Poisson’s ratio, and 2780 kg/m^3^) were considered for the analysis. Thirty-millimeter non-reflective boundaries (NRBs) were applied at the edges of the model using COMBIN14 spring-damper elements in ANSYS to eliminate the reflections from the boundaries of the plate. Figure 2 presents the application of NRBs at the boundaries. On the top and bottom surfaces, and at both ends of the plate, the COMBIN14 elements were implemented. Starting from zero in a linear pattern on the plate, the damping coefficients of the elements were varied gradually. The maximum stiffness and damping values were applied at the edges of the plate. The detailed information can be found in Ref. [19].

**Figure 3:**
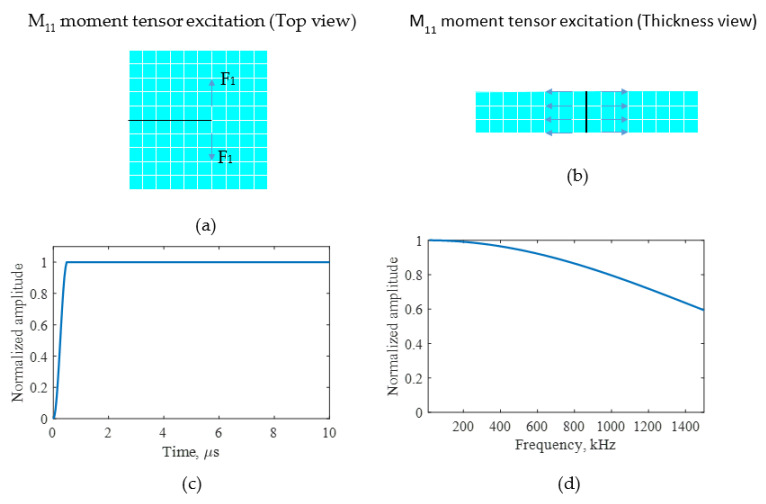
An M_11_ moment tensor excitation was applied at the crack tip for FEM simulation of AEs due to crack growth. (**a**) Top view of the M_11_ moment excitation generated using dipole forces (F_1_); (**b**) thickness view of the M_11_ moment excitation; (**c**) waveform of smooth-step excitation; (**d**) frequency spectrum of smooth-step excitation.

A 1/3 mm element size for the length and thickness of the model was used for the finite element meshing. The dipole moment excitation concept [27,42] was used for the modeling of a fatigue-crack growth source cause by a crack growth event. In this approach, the AE source caused by a fatigue-crack growth event was considered to be self-equilibrating dipole forces (M11 moment tensor) acting at the crack tip. In previous research, this source definition has been implemented for fatigue-crack growth AE numerical prediction and sensing using a PWAS sensor, and validated using experimental investigation [42]. Figure 3a,b present the modeling details of the dipole force. The top view of the dipole excitation on the meshed geometry is presented in Figure 3a and the side view of the dipole excitation is presented in Figure 3b. The M_11_ dipole excitation was defined using equal and opposite nodal forces. The time profile of the excitation was defined as a cosine-bell function with 0.5 µs as the rise time of the excitation [42]. Figure 3c,d presents the time domain of the excitation and the frequency spectrum. The acoustic waveforms generated due to the dipole excitation were obtained by performing the finite element simulation.

**Figure 4:**
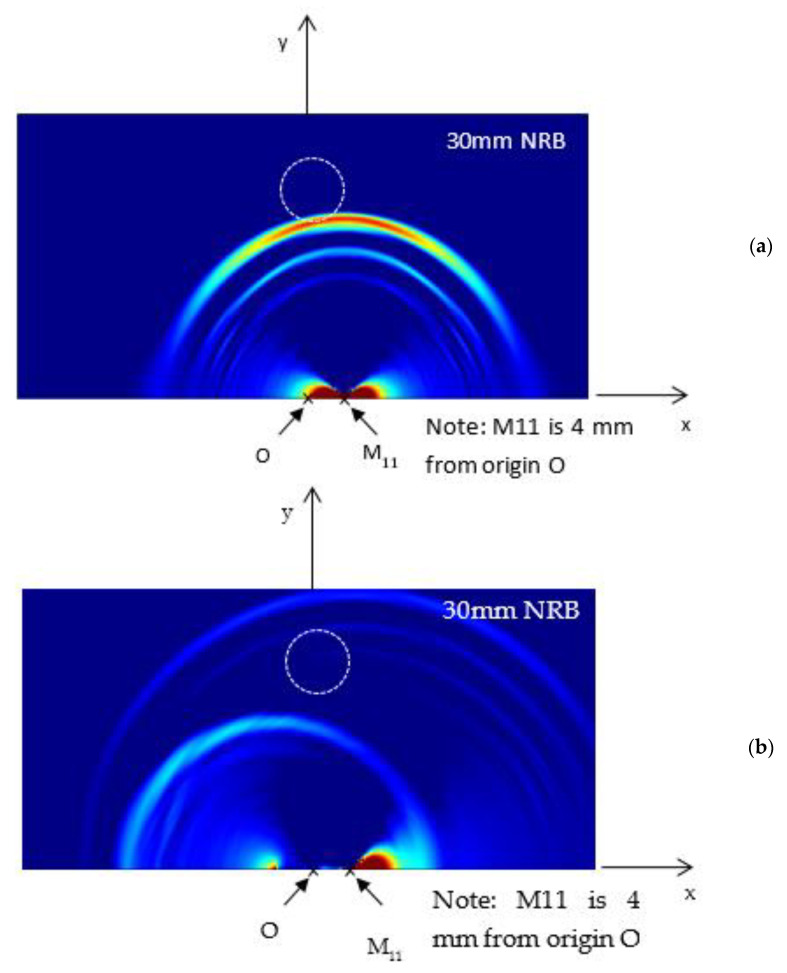
Wave propagation pattern of surface strain (*ε*_xx_ + *ε*_yy_) due to (**a**) no crack and (**b**) 8 mm crack.

Next, the FEM simulation was performed to study the wave propagation pattern due to fatigue-crack growth. The wave propagation pattern resulting from the excitation on the plate is presented in Figure 4. The surface strain (*ε*_xx_ + *ε*_yy_) extracted from the FEM simulation was plotted. Figure 4a represents the wave propagation pattern in a non-cracked specimen and Figure 4b shows the wave propagation pattern in a specimen with an 8 mm crack. As can be observed, although the excitation was the same for all simulations, the wave propagation patterns differed due to the existence of the crack. This difference is due to the resonance of AE signals originating at the crack. AE energy generated at one crack tip travels to the other tip and generates propagating waves. This causes some additional resonance and acts as an additional wave source, causing the difference in the AE wave propagation pattern compared to the no-crack situation.

**Figure 5:**
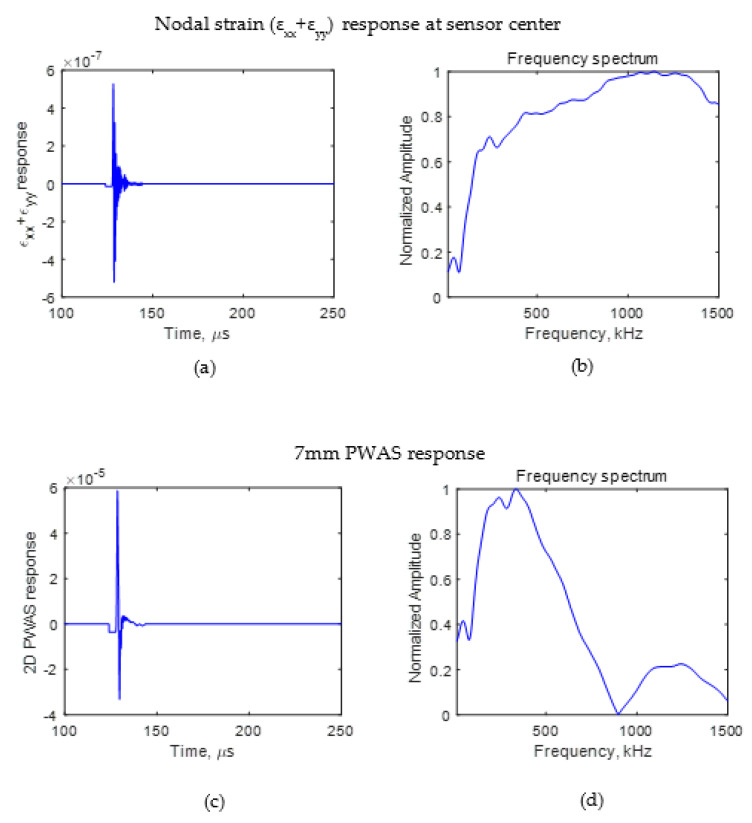
FEM simulation AE signal sensed at 25 mm in the case of no-crack. (**a**) Nodal in-plane strain response of AE signal; (**b**) frequency spectrum of nodal in-plane strain response of AE signal; (**c**) PWAS response of AE signal; (**d**) frequency spectrum of PWAS response of AE signal.

**Figure 6:**
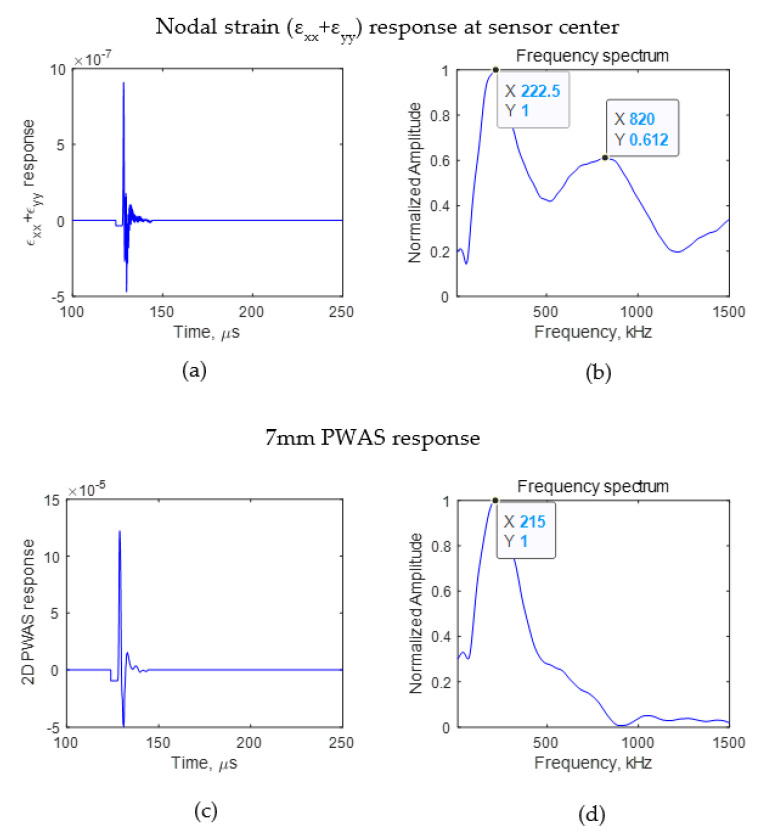
FEM simulation AE signal sensed at 25 mm. (**a**) Nodal in-plane strain response; (**b**) frequency spectrum of nodal in-plane strain response; (**c**) 7 mm PWAS response of AE signal (**d**) frequency spectrum of 7 mm PWAS response.

**Figure 7:**
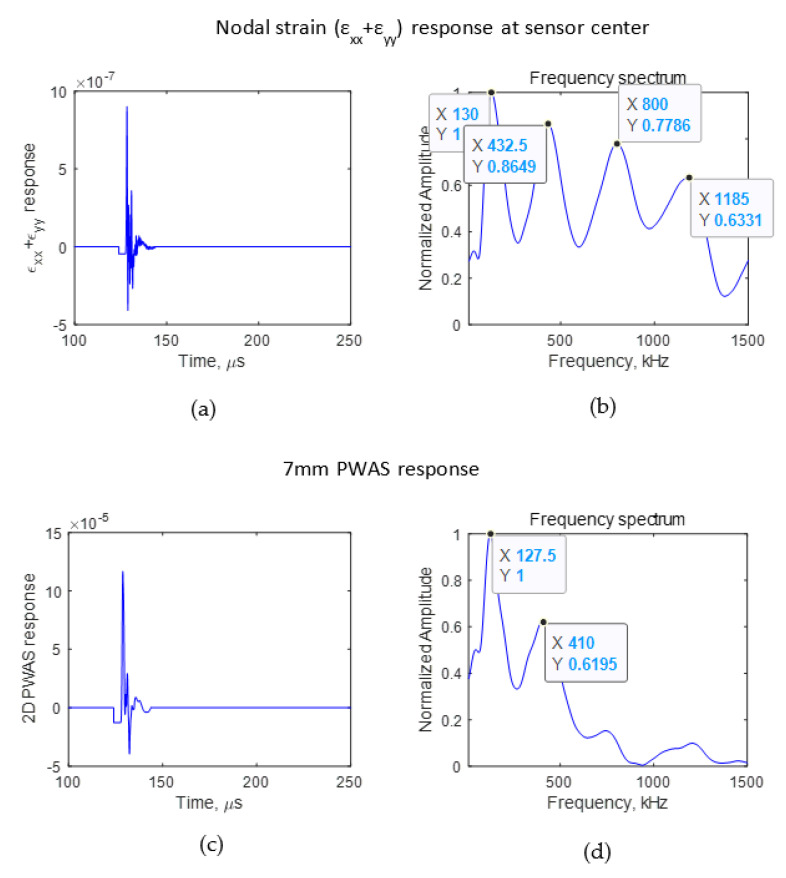
FEM simulation AE signal sensed at 25 mm in the case of an 8 mm crack. (**a**) Nodal in-plane strain response; (**b**) frequency spectrum of nodal in-plane strain response; (**c**) 7 mm PWAS response (**d**) frequency spectrum of 7 mm PWAS response.

It was seen that the crack length affects the wave propagation pattern due to an AE event. If the difference can be observed in the wave propagation pattern, the AE signals sensed using a finite size sensor also should have some differences. To identify the effect of AE signals due to the presence of a crack on an AE signal sensed using a PWAS having a finite size, the signal sensed using a sensor with a diameter of 7 mm for crack lengths of 4 and 8 mm was studied. The PWAS sensor senses the in-plane strain of the AE signal. Using Equation (2), the voltage sensed using a PWAS sensor was calculated. The PWAS was assumed to be bonded 25 mm from the crack, as presented in Figure 3. The *ε*_xx_ and *ε*_yy_ of the AE signal at the nodes where the PWAS was located were obtained from the FEM simulation. The nodal strain data were integrated numerically, and the resulting voltage response was calculated according to Equation (2). The PWAS voltage response was evaluated for the cases in which there was no crack, a 4 mm crack, and an 8 mm crack. The numerically calculated PWAS response for the no-crack case is presented in Figure 5. For 4 and 8 mm cracks, the resulting voltage response at the PWAS is presented in Figure 6 and Figure 7, respectively. Figure 5a,b, Figure 6a,b and Figure 7a,b present the nodal in-plane strain response (ε_xx_ + ε_yy_) 25 mm from the crack and the frequency spectrum. The frequency spectrum was obtained using a discrete Fourier transform with a sampling frequency of 10 MHz. Figure 5c,d, Figure 6c,d and Figure 7c,d present the 7 mm PWAS response 25 mm from the crack and the frequency spectrum. As observed in Figure 5d, Figure 6d and Figure 7d, the nodal response was modified by the PWAS resonance according to the tuning curve corresponding to the dimensions of the PWAS. The effect of the tuning curve weakened the AE signal nodal response peaks. The nodal response has specific peaks and valleys in its frequency spectrum. It should be noted that, up to 1500 kHz, the 4 mm crack has two peaks in the frequency spectrum. However, in the case of the 8 mm crack length, the nodal AE signal frequency spectrum has four peaks; that is, the number of peaks doubled as the crack length doubled. More precisely, the crack length and peaks in the frequency spectrum of the AE signal have a proportional relationship. This was also observed in the peaks of the integrated effect due to the finite-size 7 mm PWAS, which only showed a weakening effect on the peaks at higher frequencies. This proportional increment in the peaks in the frequency spectrum of the signal is due to the change in the resonance of the AE signal at the crack. Due to the change in the crack length, the resonance of the AE signal at the crack changes, which causes the variation in the frequency spectrum peak–valley pattern at the PWAS. 

## 4. Experimental Setup

### 4.1. Specimen Preparation

**Figure 8:**
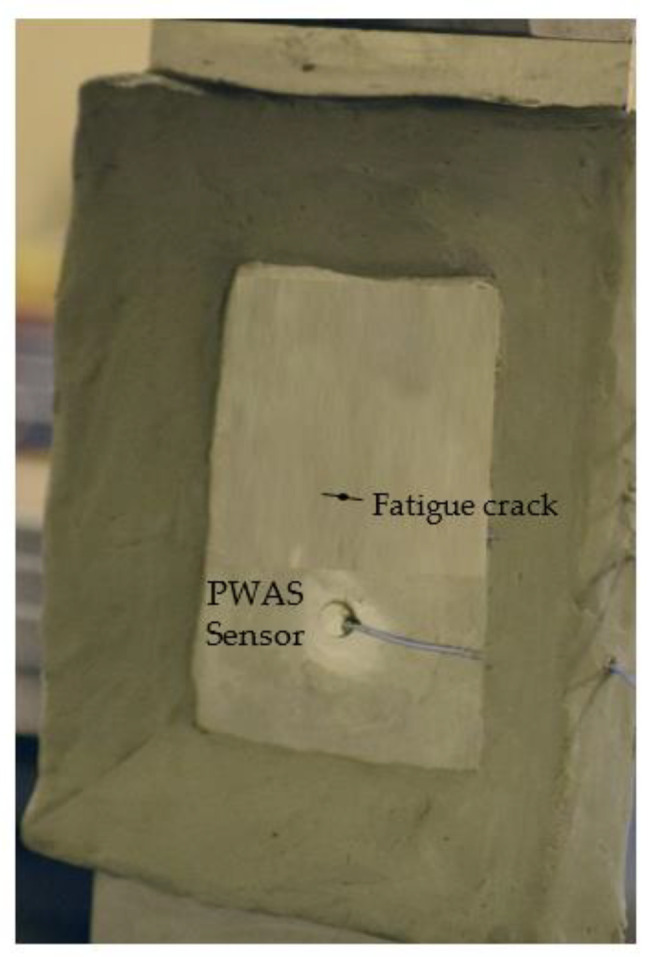
AE test specimen installed with the PWAS sensor. Non-reflective clay boundaries (NRBs) were provided on the specimen to avoid the reflection of AE signals from the specimen boundaries.

An AE experimental specimen was prepared to record AEs during crack growth in thin metallic plates. The test specimens were made of aluminum 2024-T3, a widely utilized aircraft material. A shear metal cutting machine was used to machine coupons of 103 mm width, 305 mm weight, and 1 mm thickness from a large plate of aluminum 2024-T3. The test specimens were sufficiently wide to allow a long crack to form in the specimen. Fatigue cyclic loading was performed on the test specimen by applying a fatigue load ranging from 13.85 to 1.38 kN at 10 Hz. A fatigue crack was originated from a 1 mm hole due to the continuous fatigue loading. The tip-to-tip crack length was 4 mm at 322 kcycles of fatigue loading. At crack initiation, the specimen was taken out of the MTS machine. The PWAS sensor was installed, and a non-reflective clay boundary (NRB) was implemented on the specimen. The PWAS sensor was installed at a distance of 25 mm from the crack. The NRB was applied to the specimen to eliminate AE signal reflections from the plate boundaries, ensuring clean, reflection-free AE signals were received. After the AE sensor and NRB were implemented on the specimen (Figure 8), the crack was grown by an additional 5.4 mm (until the crack length reached 9.4 mm tip to tip), while simultaneously recording the AE signals. The specimen’s wide geometry was desired for this work to ensure that the generated acoustic waves would travel a longer distance to the edges. As a result, the signals died out after reflection from the boundaries, before reaching the sensors, due to geometric spreading and material damping.

### 4.2. AE Experimental Setup

**Figure 9:**
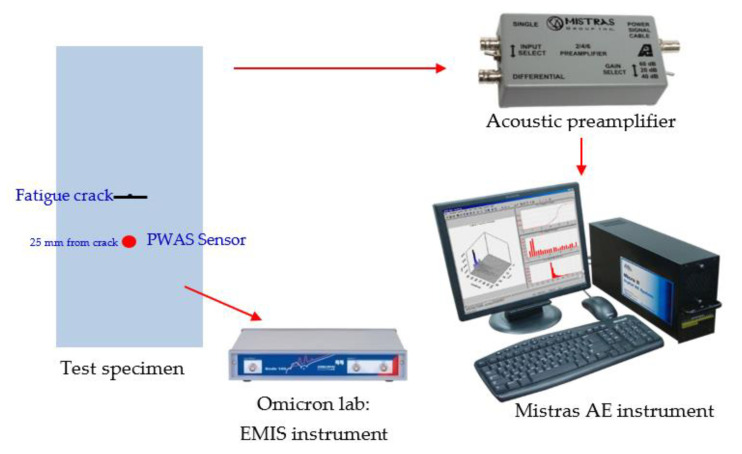
Experimental setup to capture AE signals during a fatigue-crack event.

After installing the sensor, the fatigue loading continued to grow the crack, and AE signals were simultaneously captured. The test specimen, installed with a PWAS transducer, was mounted on the MTS machine (Figure 8). The experimental setup for capturing the AE signal from a fatigue-crack growth event is presented in Figure 9. The bond quality assurance of PWAS sensors was performed periodically by electromechanical impedance spectroscopy (EMIS) [41]. AE signals during crack growth events were captured using the PWAS sensor. The sensor was connected to an acoustic preamplifier. The acoustic preamplifier is a bandpass filter that filters signals between 30 and 700 kHz. Provided with 20/40/60 dB gain (can be selected using a switch), this preamplifier operates with either a single-ended or differential sensor. In the present experiment, 40 dB gain was selected. The preamplifier was connected to the MISTRAS AE system. A sampling frequency of 10 MHz was chosen to capture any high-frequency AE signals. The timing parameters set for the MISTRAS system were peak definition time (PDT) = 200 µs, hit definition time (HDT) = 800 µs, and hit lockout time (HLT) = 1000 µs.

## 5. AE Experiment Results and Discussion

The fatigue-crack growth experiment was continued at a frequency of 2 Hz while the AE signals were captured. The coupling of the PWAS sensor to the specimen was tested using electromechanical impedance spectroscopy (EMIS) and pencil lead break tests were performed periodically. The experiment was performed using the stress intensity factor (SIF)-controlled fatigue-crack growth method. In this method, the fatigue loading was decreased when the crack length was progressed to control the stress intensity factor (SIF) at the crack tips and control the crack growth rate. This method was used to obtain a controlled crack growth. More details of this experimental method can be found in Ref. [43]. In this experiment, an additional 188 kcycles fatigue loading was performed. The non-reflective clay boundaries were used on the specimen to obtain boundary reflection-free AE signals during the experiment. 

Continuous recording of AE signals was performed during the crack growth process. The AE signals due to fatigue-crack growth were characterized from the recorded signals. It was observed that the crack growth-related AE signals changed in the frequency spectrum at various crack lengths.

**Figure 10:**
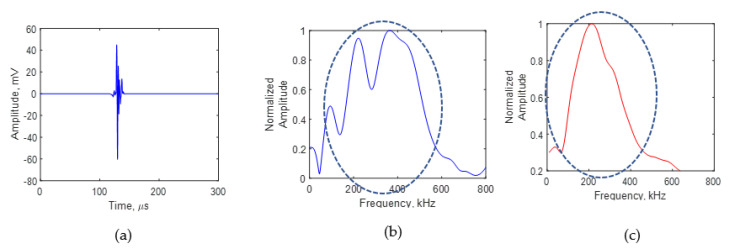
AE signal at a crack length of 4 mm: (**a**) experimental signal recorded using the PWAS sensor; (**b**) experimental frequency spectrum; (**c**) simulation frequency spectrum.

**Figure 11:**
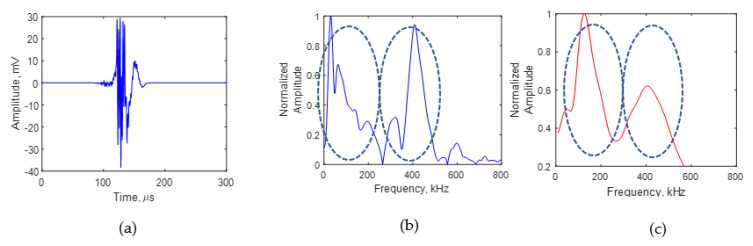
AE signal for a crack length of 8 mm: (**a**) experimental signal recorded using PWAS sensor; (**b**) experimental frequency spectrum; (**c**) simulation frequency spectrum.

A comparison of the AE signals for crack lengths of 4 and 8 mm is presented in Figure 10 and Figure 11. Figure 10 represents the AE signal for a crack length of 4 mm. Figure 10a presents the time domain of the AE signal at 4 mm. The fast Fourier transform of the time domain of the signal was performed and the frequency spectrum of the AE signal was obtained. Figure 10b is the frequency spectrum of the AE signal at 4 mm. The AE signal frequency spectrum obtained from the simulation is presented in Figure 10c. It can be seen that one major peak in the frequency spectrum was observed in the experiment signal, which was also the observation from the simulation signal. The experiment signal frequency spectrum shows minor peaks and valleys in the major peak, which are not present in the simulation. The presence of minor peaks could be due to the uncertainties in the asperities and the crack growth direction during the experiment. In the experiment, the fatigue-generated crack was not a perfectly flat crack edge as modeled in the simulation.

Figure 11 presents the experimental and simulation AE signal frequency spectrum due to crack growth for a crack length of 8 mm. Figure 11a presents the time domain of the AE signal at 8 mm. A fast Fourier transform of the experimental AE signal was performed to obtain the frequency spectrum of the signal. Figure 11b shows the frequency spectrum of the AE signal at 8 mm. The simulation AE signal frequency spectrum is presented in Figure 11c. At a crack length of 8 mm, two major peaks in the frequency spectrum can be observed in the experimental frequency spectrum. Two major peaks in the frequency spectrum of the simulation signal can also be observed. Thus, one major peak in the frequency spectrum of the AE signal received due to crack growth for a crack length of 4 mm was changed to two major peaks when the crack length was 8 mm. The simulation in Section 3 showed that the AE signal originating at the crack tip resonates at the crack before reaching the AE sensor. The AE signal resonates differently for crack lengths of 4 and 8 mm. This causes the difference in the peak–valley pattern in the frequency spectrum of the AE signal. Thus, using this novel approach, the frequency spectrum of the AE signal recorded using the PWAS can be used to approximately determine the fatigue-crack length from the AE signal recorded during fatigue-crack growth. The AE signals resonate depending on the length of the crack, which causes the peak–valley pattern in the frequency spectrum of the AE signal.

Therefore, PWAS sensors can be effectively used to determine the length of the crack from AE signals. This paper presented the proof of concept of the method from experimental results and simulation. The high-frequency resonances excited in the crack by the energy released when the crack advances contain useful information about the crack. These high-frequency resonances cause the frequency spectrum of the AE signal to change. The working principle of the PWAS sensor explained in Section 2 is also an important factor in utilizing this phenomenon for crack length-detection. The surface-mounted PWAS sensors record in-plane strain due to AEs. This research showed that the in-plane strain measured using a PWAS reflects the length of the crack in the frequency spectrum of the AE signal. Therefore, the working principle of the sensor, and the measurements from the AEs using this working principle, are important deciding factors. Hence, the use of the PWAS for AE measurement in this study was crucial.

## 6. Summary, Conclusions, and Future Work

### 6.1. Summary

The paper presented a novel method for correlating crack length and AE signals during the growth in fatigue cracks. In this novel method, the frequency spectrum of the AE signal was studied to obtain crack length information. In this paper, the theoretical equation for sensing the AE signal using a PWAS sensor was first derived. Next, FEM analysis was performed to simulate the AE wave propagation during a fatigue-crack growth event. A dipole excitation at the crack tip was applied to define the fatigue-crack growth AE source and simulate the crack growth AE event. The voltage response of a PWAS sensor located 25 mm from the crack was calculated using the FEM simulation strain field and theoretically derived equations. Based on the simulation results, a specific peak–valley pattern for the AE signal frequency spectrum was observed, and was dependent on the crack length. A novel SIF-controlled fatigue-crack growth experiment was performed to record AE signals during fatigue-crack growth. AE signals of fatigue-crack growth were recorded when the crack lengths were 4 and 8 mm. The frequency spectrum of the AE signals was studied in a frequency range of 0 to 700 kHz.

### 6.2. Conclusions

The AE signals during fatigue-crack growth were observed to vary with an increase in crack length. Due to the fatigue-crack growth, AE signals are generated at the crack tip. The generated AE energy resonates with the crack, forming a standing wave pattern. The fatigue-crack length influences this standing wave pattern. The frequency spectrum of AE signals recorded with the PWAS transducer is affected by this phenomenon. Based on the FEM numerical analysis of a 7 mm PWAS sensor installed 25 mm from the crack, the AE signal for a crack length of 4 mm had only one significant peak in the frequency spectrum up to 700 kHz. However, for a crack length of 8 mm, two significant peaks were observed in the frequency spectrum of the simulated AE signal. Similar phenomena were also observed in the experimental investigation. Thus, the frequency spectrum of the AE signal caused by fatigue-crack growth can be used to evaluate the length of the crack from the AE signals.

### 6.3. Future Work

In the future, an analytical expression to correlate the crack length and AE signal could be developed.
